# Decoding the Implications of Zinc in the Development and Therapy of Leukemia

**DOI:** 10.1002/advs.202412225

**Published:** 2025-01-30

**Authors:** Bo Zhu, Chunhao Yang, Siqi Hua, Kaiqiang Li, Pengyou Shang, Zhonghua Li, Wei Qian, Shunkang Xue, Qi Zhi, Zichun Hua

**Affiliations:** ^1^ School of Biopharmacy China Pharmaceutical University Nanjing 211198 China; ^2^ Changzhou High‐tech Research Institute of Nanjing University and Jiangsu TargetPharma Laboratories Inc. Changzhou 213164 China; ^3^ Department of Radiology Affiliated Hospital of Nanjing University of Chinese Medicine Nanjing 210029 China; ^4^ The State Key Laboratory of Pharmaceutical Biotechnology School of Life Sciences Nanjing University Nanjing 210023 China; ^5^ Faculty of Pharmaceutical Sciences Xinxiang Medical University Xinxiang 453003 China

**Keywords:** Immunotherapy, Leukemia, onco‐fusion proteins, trace elements, zinc homeostasis

## Abstract

Zinc plays a central role in the hematological development. Therapeutic interventions with zinc are shown to improve the health status of patients with malignancies by stimulating the immune system and reducing side effects. Despite the abnormal zinc homeostasis in leukemia, the role and mechanisms of zinc signaling in leukemia development remain poorly understood. Recently, some important breakthroughs are made in laboratory and clinical studies of zinc in leukemia, such as the role of zinc in regulating ferroptosis and the effects of zinc in immunotherapy. Zinc‐based strategies are urgently needed to refine the current zinc intervention regimen for side‐effect free therapy in chemotherapy‐intolerant patients. This review provides a comprehensive overview of the role of zinc homeostasis in leukemia patients and focuses on the therapeutic potential of zinc signaling modulation in leukemia.

## Graphical abstract

Zinc's pivotal role in hematological development and its therapeutic potential in leukemia are explored. This review delves into the complex interplay of zinc signaling in leukemia progression and treatment. It highlights recent advancements, such as zinc's regulation of ferroptosis and its impact on immunotherapy, underscoring the need for zinc‐based strategies to enhance leukemia therapy, particularly for patients intolerant to chemotherapy.

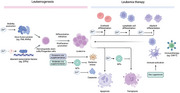

## 1 Introduction

Leukemia resulting from the dysregulation of hematopoietic stem cells is classified into several types, including acute myeloid leukemia (AML), acute lymphoblastic leukemia (ALL), chronic myeloid leukemia (CML), and chronic lymphocytic leukemia (CLL).^[^ ^1^, ^2^, ^3^, ^4^, ^5^ ^]^ Leukemia poses significant risks for its high frequency in children, and pediatric patients are low tolerant to the toxic effects of the standard chemotherapy regimen.^[^ ^6^ ^]^ Side effects caused by existing standard treatment regimens increase the mortality rate of children with leukemia.^[^ ^7^ ^]^ A balanced adjuvant is urgently needed in leukemia therapy, which requires the ability to reduce toxicity and increase therapeutic efficacy.

**Figure 1:**
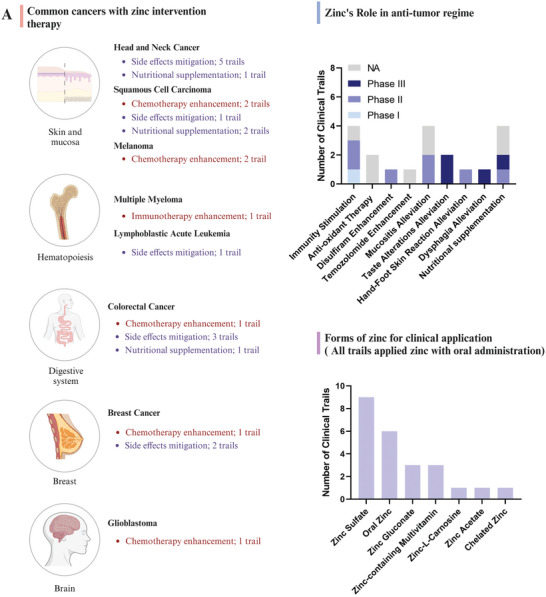
Clinical applications of zinc in cancer therapy. This figure summarizes ongoing clinical trials investigating zinc‐related interventions for tumor therapy, as recorded on ClinicalTrials.gov (as of July 10, 2024). A) Different types of cancer being targeted with zinc therapy. B) Phases of each clinical trial and highlights the role of zinc within these trials. C) Different forms of zinc used in the clinical trials.

Zinc serves as a critical trace element, and is clinically used in cancer therapy as an adjuvant for alleviating side effects or enhancing chemotherapy (**Figure** **1**). We emphasized the immune‐stimulating ability of zinc is widely presented in different carcinomas, suggesting the potential role of zinc to modulate hematologic malignancies development. Leukemia cells are more sensitive to both zinc deficiency and zinc overload than peripheral blood lymphocyte cells, suggesting an essential role for zinc in leukemia therapy.^[^ ^8^, ^9^ ^]^ Additionally, clinical studies reported the reduced serum zinc levels in leukemia (**Table** **1**), further supporting the association between zinc dysregulation and leukemia development. While zinc serves as a critical trace element involved in the development of physiological functions, especially hematopoiesis, zinc is well tolerated in pediatric practice.^[^ ^10^, ^11^ ^]^ Clinical evidence has demonstrated that zinc supplementation can improve oropharyngeal mucositis in childhood acute leukemia, suggesting the potential side‐effect mitigating efficacy of zinc intervention therapy for children with leukemia.^[^ ^12^ ^]^ Therefore, the exploration of zinc‐based strategies for leukemia intervention therapy may benefit chemotherapy‐intolerant patients, especially in children.

**Table 1:** Altered Zinc Levels in Leukemia Patients.

References	Year	Sample size	Changes in zinc levels	Other information
[13]	2018	Acute leukemia (AL) (*n* = 1122); Non‐leukemia (*n* = 1372)	Lower serum zinc levels were observed in the AL group compared with the control group.	Lower serum selenium levels were observed in the AL group compared with the control group.
[14]	1989	AL (45); Control (*n* = 30)	Serum zinc levels were lower in different stages of acute leukemia patients compared with the healthy group.	Serum nickel level was higher in the onset phase and futile treatment acute leukemia patients compared with the healthy group.
[15]	2011	AL (*n* = 42); Control (*n* = 40)	Lower serum zinc levels were observed in the AL group compared with the control group.	i. Lower serum magnesium and manganese levels were observed in the AL group compared with the control group; ii. Higher serum copper, lead, and cadmium levels were observed in the AL group compared with the control group; iii. Serum iron and cobalt levels had no significant difference between the leukemia group and the control group.
[16]	2000	ALL (*n* = 20); Lymphoma (*n* = 26); healthy group (*n* = 12)	i. Serum zinc levels in lymphoma patients were significantly lower than healthy group; ii. Hair zinc levels in both lymphoma and ALL patients were significantly lower than in the healthy group.	Hair magnesium levels in T‐ALL patients were significantly lower than in the healthy group.
[17]	1999	ALL (*n* = 23) and ANLL (*n* = 23)	Serum zinc levels in children with leukemia were significantly lower than in the control group, without being overtly malnourished.	Serum copper levels in children with leukemia were significantly higher than in the control group.
[18]	2010	ALL (*n* = 26); Control (*n* = 10)	i. Serum zinc level in ALL was significantly lower than in the patients before treatment; ii. Serum zinc level in ALL that undergoing XV chemotherapy regime was significantly higher than in the patients before treatment;	The XV chemotherapy treatment causes CD95 upregulation and Bcl‐2 downregulation, suggesting the potential pro‐apoptosis efficacy.
[19]	2018	ALL (*n* = 30);	Serum zinc level in ALL after chemotherapy treatment was significantly higher than the patients before treatment.	i. Serum copper level in ALL after chemotherapy treatment was significantly lower than the patients before treatment; ii. Cu/Zn SOD activity in ALL after chemotherapy treatment was significantly higher than in the patients before treatment.
[20]	2019	ALL (*n* = 32); AML (*n* = 16); Control (*n* = 36)	Serum zinc levels in both ALL and AML were significantly lower than in the control group.	i. Serum selenium levels in both ALL and AML were significantly lower than the control group; ii. Serum copper levels in both ALL and AML were significantly higher than in the control group.
[21]	1991	ALL (*n* = 33); AML (*n* = 3); Lymphoma (*n* = 5)	i. Serum zinc levels in both AL and lymphoma during induction treatment were significantly lower than in controls; ii. Urine zinc levels in both AL and lymphoma during induction treatment were significantly higher than controls; iii. Hair zinc levels in both AL and lymphoma at the onset phase or during induction treatment were significantly higher than in controls.	There was no significant difference in zinc levels in cerebrospinal fluid between the experimental and control groups.
[22]	2021	ALL (*n* = 36)	i. Serum zinc levels in ALL undergoing chemotherapy were significantly lower than those before treatment; ii, Patients undergoing high‐risk chemotherapy regimes presented lower serum zinc levels than the patients treating standard‐risk chemotherapy regimes.	Serum copper, manganese, magnesium, chromium, and iron levels in ALL undergoing chemotherapy were significantly lower than those before treatment.
[23]	2006	ALL (*n* = 45)	Serum zinc levels in ALL after chemotherapy and radiotherapy had no significant difference compared with the patients before treatment.	Serum copper level in ALL after chemotherapy was significantly lower than the patients before treatment.
[24]	2006	ALL (*n* = 7); ANLL (*n* = 34); CML (*n* = 9); Control (*n* = 20)	Serum zinc levels in both AL and CML were significantly lower than in the control group.	i. Serum selenium level in AL was significantly lower than the control group; ii. Serum copper levels in both AL and CML were significantly higher than in the control group; iii. Cu/Zn SOD activity in AL was significantly higher than the control group;
[25]	2011	AML (*n* = 30)	Serum zinc levels in AML patients with chemotherapy were significantly higher than the patients before treatment.	The serum Cu/Zn ratio in AML patients with chemotherapy was significantly higher than the patients before treatment.
[26]	1987	CLL stage 0–2 (*n* = 40); CLL stage 3–4 (*n* = 10); Control (*n* = 100)	i. Serum zinc levels both in CLL stage 0–2 and stage 3–4 were significantly lower than the control group; ii. Serum zinc level in CLL stage 0–2 was not significantly different from CLL stage 3–4.	i. Serum selenium level in CLL was not significantly different from to control group; ii. Serum copper level in CLL was significantly higher than the control group; iii. Serum calcium level in CLL stage 3–4 was significantly lower than the control group; iv. Cu/Zn ratio was significantly lower in healthy subjects compared with hematological malignancies patients.
[27]	1995	Lymphoma (*n* = 17); acute leukemia (*n* = 15); chronic leukemia (*n* = 12); healthy group (*n* = 96)	i. Serum zinc levels in hematological malignancy patients were significantly lower than healthy group; ii. Serum zinc levels in patients who died in the following 13 months were significantly lower than those who still survived.	The Cu/Zn ratio was significantly lower in healthy subjects compared with hematological malignancies patients.

To delve deeper into the therapeutic potential of zinc in leukemia, this review presents a summary of the characteristics of zinc homeostasis in leukemia patients and discusses the possible mechanisms by which zinc signaling may regulate leukemia progression. We aim to offer innovative insights into the clinical application of zinc and to foster new perspectives for future research in zinc‐based therapies.

## 2 Cellular and Systemic Physiology of Zinc

The role of zinc in biological systems was first identified in 1869 when it was found to be essential for the growth of Aspergillus niger.^[^ ^28^ ^]^ Dr. Ananda Prasad's seminal work established zinc as an essential nutrient for human health in 1963.^[^ ^28^ ^]^ Nowadays, zinc is recognized as the second most abundant trace element in the human body, with ≈60% stored in muscle, 30% in bone, and ≈5% in skin and liver.^[^ ^29^ ^]^ Zinc absorption occurs primarily in the duodenum and jejunum, where it crosses the intestinal epithelium as free ions.^[^ ^29^ ^]^ In the circulatory system, ≈80% of zinc is loosely bound to albumin, while ≈20% is tightly bound to α2‐macroglobulin, facilitating its transport and storage.^[^ ^29^, ^30^ ^]^

**Figure 2:**
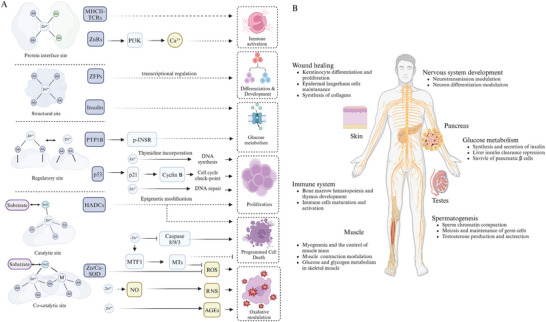
Cellular and Systemic Physiology of Zinc. Zinc binds to four distinct sites: regulatory and catalytic sites, which influence the activity of zinc‐dependent enzymes based on zinc concentration; structural sites, such as those in zinc finger proteins, whose expression is regulated by zinc homeostasis but whose activity is not; and protein interface sites, which modulate receptor‐ligand affinity (e.g., TCRs‐MHCII), affecting cellular communication. Zinc modulates cell fate through enzyme activity, transcription factors, and signaling pathways, influencing processes like PCD differentiation, and proliferation. It is crucial for maintaining redox balance and glucose metabolism, and is essential for the development of the immune, nervous, and reproductive systems (particularly spermatogenesis). Additionally, zinc plays a vital role in muscle regeneration and skin wound healing. Abbreviations: ZnRs, zinc‐sensing receptors; ZFPs, zinc finger proteins; PTP1B, protein tyrosine phosphatase 1B; p‐INSR, phosphorylated insulin receptor; HDACs, histone deacetylases; ROS, reactive oxygen species; RNS, reactive nitrogen species; AGEs, advanced glycation end products.

Proteins interacting with zinc usually exert their function as enzymes or transcription factors.^[^ ^10^ ^]^ Four types of classical zinc‐binding sites have been identified as structural site, regulatory site, catalytic site and protein interface site.^[^ ^31^ ^]^ Changes in zinc levels may alter the expression, affinity, structure or activity of these zinc‐binding proteins, regulating signaling and thereby modulating physiological functions such as proliferation, programmed cell death (PCD), differentiation and redox balance^[^ ^10^ ^]^ (**Figure** **2**).

### 2.1 Intracellular Zinc Distribution

Upon entering cells, the majority of zinc ions bind to proteins, acting as catalytic sites or structural components, and are distributed throughout the cytoplasm (50%), nucleus (30%), and membrane (10%).^[^ ^32^, ^33^ ^]^ A small fraction of zinc is present as free ions, with concentrations ranging from picomolar to nanomolar in the cytoplasm and within intracellular organelles such as the Golgi apparatus, endoplasmic reticulum (ER), and mitochondrial matrix.^[^ ^34^, ^35^ ^]^ Specialized compartments like synaptic vesicles and insulin granules have high levels of free zinc (≈100 µm).^[^ ^36^, ^37^ ^]^ Under pathological conditions, the intracellular distribution of zinc alters and even leads to the formation of zincosomes.^[^ ^38^ ^]^ Similarly, zinc levels in ER and nucleus can fluctuate dramatically in response to pH stimuli, potentially contributing to the generation of zinc waves.^[^ ^38^ ^]^

### 2.2 Zinc Homeostasis‐Related Proteins

Cellular zinc homeostasis is primarily regulated by two families of transporters: the zinc transporters (SLC30A/ZnTs) and the Zrt/Irt‐like proteins (SLC39A/ZIPs). The ZnT family, consisting of 10 members, controls the efflux of free zinc from the cytoplasm to organelles or extracellular space.^[^ ^29^ ^]^ Three aspartic acids and one histidine on transmembrane helices II and V form an intramembranous zinc‐binding site that is critical for zinc transport.^[^ ^29^, ^39^ ^]^ Recently, TMEM163 was identified as a novel member of the ZnT family capable of binding and transporting zinc ions from the cytoplasm, with key binding sites being conserved aspartate residues (D124A/D128A and E286K).^[^ ^40^ ^]^ ZIP transporters are not exclusively involved in zinc transport; they also facilitate the movement of iron, manganese, copper, and cadmium.^[^ ^41^ ^]^ The ZIP family consists of 14 members, characterized by eight transmembrane domains, with transmembrane helix V, playing a critical role in zinc influx into the cytoplasm.^[^ ^29^ ^]^ In addition, transient receptor potential channels and glutamate receptor channels contribute to zinc transport, albeit with less specificity.^[^ ^42^, ^43^ ^]^ Metallothioneins (MT), proteins that bind intracellular zinc, serve as a reservoir and are essential for zinc homeostasis maintaining.^[^ ^44^ ^]^ Moreover, the first zinc chaperone, ZNG1, was identified in 2023, that responds to targeting and inserting zinc into specific proteins, particularly under zinc‐deficient conditions.^[^ ^45^ ^]^

## 3 Abnormal Zinc Homeostasis in Leukemia

### 3.1 Disruption of Systematic Zinc Distribution

Altered serum zinc levels have been clinically observed since 1987 and have emerged as a potentially significant prognostic biomarker for cancer outcome.^[^ ^46^ ^]^ Lower serum zinc levels (SZL) tended to occur in leukemia patients and were associated with higher mortality rates.^[^ ^47^ ^]^ A more pronounced reduction of SZL is seen in acute leukemia compared to chronic leukemia.^[^ ^27^ ^]^ However, no clear distinction of SZL was observed between different types and stages of leukemia.^[^ ^26^ ^]^ Decreased SZL combined with elevated serum copper levels is emerging as a potential biomarker for predicting leukemia development.^[^ ^23^, ^26^ ^]^ The serum Cu/Zn ratio may also be helpful in the diagnosis and prognosis prediction of leukemia.^[^ ^27^, ^48^, ^49^ ^]^ However, changes in serum Cu/Zn ratio are not leukemia specific, suggesting that it may be used as a complementary marker for hematologic malignancies^[^ ^50^ ^]^

Reduced serum zinc is common in leukemia patients (Table 1), while abnormal accumulation of zinc has been found in leukemia cells.^[^ ^51^ ^]^ Similarly, zinc in the urine and liver is elevated in leukemia compared to healthy controls.^[^ ^52^, ^53^ ^]^ An increasing trend in hair zinc levels has also been observed in ALL patients.^[^ ^52^ ^]^ Therefore, we hypothesized that the decrease in SZL in leukemia patients is related to impaired zinc distribution rather than systemic zinc deficiency.

Notably, both urinary and hair zinc levels in ALL patients normalized after continued treatment, suggesting that factors such as treatment regimen affect zinc distribution in leukemia.^[^ ^52^ ^]^ Extensively, other factors including gender, geographic region, and genetic factors, may also contribute to the abnormal distribution of zinc in patients with leukemia.

### 3.2 Abnormal Expression of Zinc Homeostasis‐Related Proteins in Leukemia

**Figure 3:**
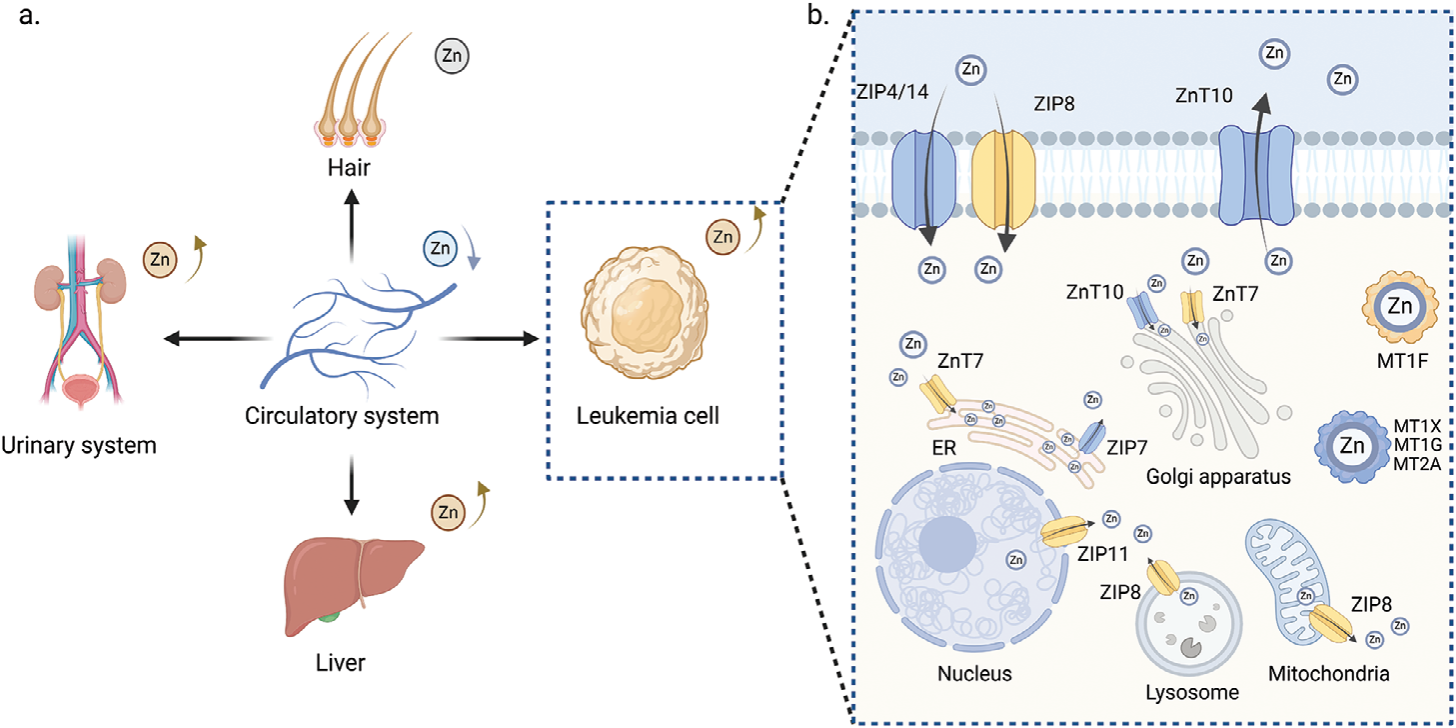
Systemic and Cellular Zinc Distribution in Leukemia Patients. In leukemia, serum zinc levels are typically low, while concentrations in the liver, urine, and tumor cells are elevated. In particular, zinc transporters such as ZIP4, ZIP7, ZIP14, ZnT10, and certain metallothioneins (MT1G, MT1X, MT2A) are downregulated, whereas ZIP8, ZIP11, ZnT7, and MT1F are upregulated. This expression pattern suggests increased zinc levels in the cytoplasm and endoplasmic reticulum, with decreased levels in the nucleus, lysosome, and mitochondria of leukemia cells compared to normal cells. In addition, variations in zinc transporter expression may influence zinc distribution in organelles such as the Golgi apparatus, although the specifics remain unclear.

The expression pattern of zinc homeostasis‐related genes changes during leukemia development and these changes may be subtype specific. High expression levels of ZIPs and ZnTs were observed in acute promyelocytic leukemia (APL) cells, providing the basis for the dramatic zinc fluctuation in APL.^[^ ^51^ ^]^ The recently identified zinc import channel Glur3 was found to be overexpressed in T‐ALL and T‐lymphoma cells, leading to a potential enhancement of zinc uptake.^[^ ^43^, ^54^, ^55^ ^]^ Bioinformatic analyses based on the TCGA and GTEx projects highlight increased expression of ZIP8, ZIP11, and ZnT7 and decreased expression of ZIP4, ZIP7, ZIP14, ZnT3, and ZnT10 in patients with AML.^[^ ^56^ ^]^ The different location of zinc transporters was associated with the specific expression pattern, suggesting the potential subcellular zinc distribution in AML, which requires further exploration (**Figure** **3**). For example, overexpression of ZIP8 may enhance zinc transport from mitochondria or lysosomes to the cytoplasm. High expression of ZnT7 and low expression of ZIP7 suggest ER zinc accumulation in leukemia cells. Reduced nuclear zinc and increased cytoplasmic zinc in leukemia cells may be partially explained by the high expression of ZIP11.^[^ ^51^, ^57^ ^]^

The specific roles of different MT family members are currently a hot spot in zinc biology research. Downregulation of MT1G, MT1X, and MT2A and upregulation of MT1F and MTF1 have been reported in AML cells,^[^ ^56^ ^]^ suggesting a reduced capacity of AML cells to tolerate zinc fluctuations. As a sensor of intracellular free zinc, the role of MTF1 in leukemia is underestimated and deserves further in‐depth study. Since the expression levels of MTs, ZIPs, and ZnTs have a feedback regulatory relationship with intracellular zinc signaling, we suggest that future studies of changes in the expression of these genes should be combined with intracellular zinc levels.

## 4 Role of Zinc in Leukemia Progression

### 4.1 The Impact of Zinc Levels on Leukemia Prognosis

Clinically, lower SZL correlates with a higher risk of leukemia.^[^ ^58^ ^]^ However, dietary zinc deficiency, rather than zinc supplementation, exerted an anti‐tumor effect in animal models.^[^ ^59^ ^]^ There is no evidence that low SZL promotes the development of leukemia, but it represents a systematic zinc deficiency in patients, which is associated with serious side effects and a high mortality rate.^[^ ^58^ ^]^ Zinc supplementation significantly reduces the proportion of high‐risk ALL patients by reducing infections and improving patients' quality of life.^[^ ^60^ ^]^ Nowadays, zinc supplements are used as a nutritional agent rather than a drug, mainly for zinc‐deficient leukemia patients. Excessive zinc intake is most common individuals with zinc deficiency, which has been associated with increased hospitalization rates in male patients with CLL and CML.^[^ ^61^ ^]^ Reducing the proleukemic effects of zinc while taking advantage of its nutritional support role, or ameliorating the side effects of zinc deficiency while taking advantage of zinc chelation‐based strategies, are the focus of future research.

### 4.2 The Interplay between Zinc Signaling and Leukemogenesis

As a second messenger, free zinc influences tumor cell death and differentiation.^[^ ^62^ ^]^ Specifically, zinc signaling exerts different physiological functions with different residence times.^[^ ^62^ ^]^ The rapid fluctuation of zinc is referred to as fast zinc signaling (FZS), which occurs over seconds to minutes and regulates the phosphorylation status of signaling molecules, such as protein tyrosine phosphatases and kinases, for intracellular signaling modulation.^[^ ^63^ ^]^ Late zinc signaling (LZS) occurs over hours and involves the regulation of protein transcription and degradation, changes in zinc homeostasis proteins, and zinc enzymes.^[^ ^64^ ^]^ The distinct effects of zinc signaling and zinc‐related proteins confer unique efficacy to FZS and LZS.

#### 4.2.1 Fast Zinc Signaling Promotes Leukemia Differentiation

Zinc signaling within seconds to minutes has been identified to be primarily involved in immune activation in immune cells.^[^ ^63^ ^]^ A nuclear zinc spark has also been observed in PMA‐induced APL cells, which is required for myelomonocytic differentiation.^[^ ^65^ ^]^ In a previous study, we observed activation of the extracellular signal‐regulated kinase (ERK)1/2 pathway after N, N, N', N'‐tetrakis (2‐pyridylmethyl) ethylenediamine (TPEN)‐mediated zinc depletion within 1 h, peaking after 3 h of treatment, presumably due to changes in zinc levels over minutes.^[^ ^66^ ^]^ Given that ERK1/2 has been implicated in leukemia cell proliferation, the activation of ERK1/2 by TPEN suggests that the zinc wave may exert an anti‐tumor effect by promoting differentiation and inhibiting proliferation.^[^ ^67^ ^]^

#### 4.2.2 Degradation of Oncofusion Proteins by Zinc Deficiency

**Figure 4:**
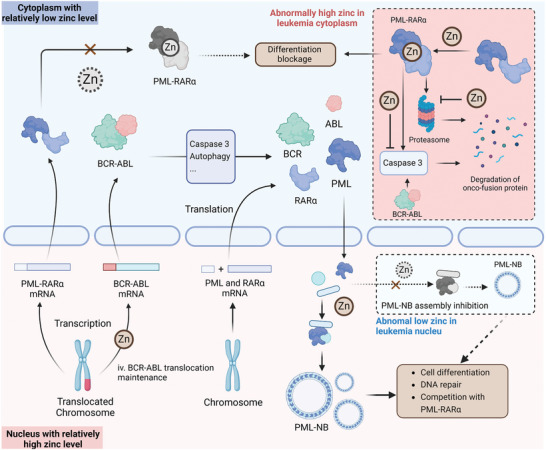
The Role of Zinc in Stabilizing Oncogenic Fusion Proteins in Leukemia. In the cytoplasm, zinc contributes to the formation and stability of the PML‐RARα fusion protein (i). It also inhibits caspase‐3 activity, which is essential for the degradation of this protein (ii). In the nucleus, zinc promotes the assembly of PML nuclear bodies (PML‐NBs), where PML influences differentiation and disrupts PML‐RARα formation (iii). In addition, nuclear zinc is critical for BCR‐ABL transcription (iv). Dysregulated zinc levels, characterized by increased cytoplasmic and decreased nuclear zinc, may increase the stability of oncogenic fusion proteins, thereby facilitating leukemia pathogenesis.

Abnormal chromosomal translocations lead to the formation of oncofusion proteins that disrupt hematopoiesis, which is considered to be the major pathogenic factor in leukemia. Zinc depletion has been shown to downregulate the expression of oncofusion proteins such as promyelocytic leukemia protein‐retinoic acid receptor alpha (PML‐RARα) and breakpoint cluster region‐abelson (BCR‐ABL) (**Figure** **4**), suggesting an essential role for zinc homeostasis in leukemogenesis.^[^ ^51^, ^68^, ^69^ ^]^

The chromosomal translocation t(15;17)(q22;q12), which occurs in more than 10% of AML patients, results in the fusion of PML and RARα and is recognized as a hallmark of APL.^[^ ^70^ ^]^ The formation of PML‐RARα blocks transcription stimulated by nuclear receptor signaling for differentiation.^[^ ^71^ ^]^ Zinc binds directly to cysteine residues of the zinc finger motif for PML‐RARα stabilization, which can be replaced by arsenic and lead to PML‐RARα degradation.^[^ ^72^ ^]^ While PML‐RARα resulting from chromosomal translocations is likely to be ribosomally translated in the cytoplasm and to build the correct conformation in the ER, higher levels of zinc in the cytoplasm and ER can be attributed to the stabilization of PML‐RARα. Zinc in the cytoplasm prevents the degradation of PML‐RARα and BCR‐ABL by suppressing the activity of caspase 3 and autophagy.^[^ ^51^ ^]^

Zinc also exists in the N‐terminal region of promotes the small ubiquitin‐like modifier (SUMO) interacting motif (SIM), promoting SUMO to interact with proteins with the SIM region, especially in phosphorylation. Zinc supplementation was shown to promote the SUMO‐SIM interaction‐based recruitment of nuclear proteins to PML for the assembly of PML nuclear bodies (PML‐NBs), the unit for PML physiological functions.^[^ ^73^ ^]^ While PML‐RARα disrupts the integrity of PML‐NBs, the re‐establishment of these nuclear bodies is critical for DNA damage repair in APL pathogenesis, suggesting a competitive relationship between PML‐NBs and PML‐RARα.^[^ ^74^, ^75^ ^]^ Therefore, the low nuclear zinc in leukemia is predicted to inhibit the formation of PML‐NBs and thus maintain the stability of PML‐RARα. Notably, nuclear zinc was also found to be essential for BCR‐ABL transcriptional activity and mRNA stability, suggesting the potential benefit of low nuclear zinc in BCR‐ABL+ CML or ALL.^[^ ^68^ ^]^

#### 4.2.3 Regulation of Redox Balance and Programmed Cell Death by Zinc

**Figure 5:**
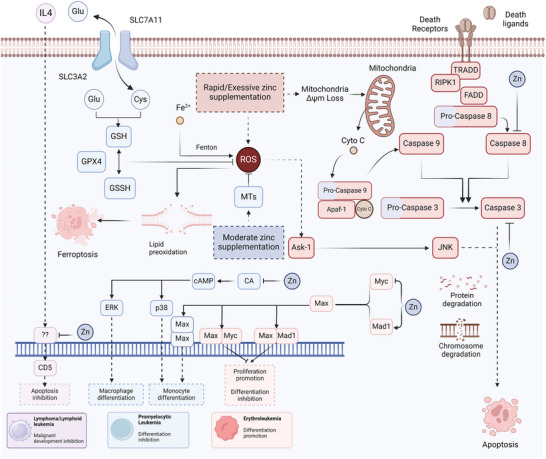
The Role of Zinc in Regulating Leukemia Cell Death and Differentiation. Under normal conditions, zinc protects against apoptosis and ferroptosis by improving DNA repair, inhibiting caspase activity, and reducing oxidative stress. However, at toxic levels, zinc can induce apoptosis in leukemia cells primarily through oxidative stress pathways. Zinc typically inhibits cAMP‐mediated differentiation in myelomonocytic leukemia by suppressing adenylate cyclase activity, which may compromise therapeutic responses. Conversely, zinc has been shown to promote differentiation in lymphoid leukemia and decrease the expression of the malignant marker CD5, suggesting a potential therapeutic benefit. In erythroleukemia, zinc affects the balance of the Myc/Mad network, promoting differentiation under certain conditions.

Oxidative stress stimulates the proliferation of leukemic cells, which favors the development of leukemia.^[^ ^76^ ^]^ To counteract the oxidative environment, elevated reductases are also observed in leukemia patients, suggesting a reductase dependency for leukemia.^[^ ^77^ ^]^ Therefore, the effect of zinc on redox balance and PCD is essential for leukemia progression (**Figure** **5**).

Without the multivalent states, zinc does not directly participate in redox reactions, but promotes the activity of zinc‐dependent reductases such as Zn/Cu‐SOD for redox modulation. Zinc can also play an antioxidant role by upregulating the expression of disulfide bond‐rich MTs.^[^ ^78^ ^]^ In leukemia, MT1 M and MT2A prevent the newly identified PCD ferroptosis, and MT2A, MT1G, MT1X, and MT3 prevent apoptosis, mainly by alleviating oxidative stress.^[^ ^79^, ^80^, ^81^, ^82^, ^83^ ^]^ Low‐dose zinc supplementation also promotes DNA repair and protects leukemia cells from apoptosis induced by topoisomerase inhibitors, DNA synthesis inhibitors, or UV radiation.^[^ ^84^, ^85^, ^86^, ^87^ ^]^ Zinc also abolishes the activity of caspase 3/6/7/8 at sub‐micromolar concentrations, preventing both intrinsic and extrinsic apoptosis under moderate zinc supplementation.^[^ ^88^, ^89^ ^]^

Intense zinc fluctuation activates the oxidative stress pathway such as Ras/ERK, leading to excessive production of reactive oxygen species (ROS) and nitric oxide (NO).^[^ ^90^, ^91^ ^]^ Meanwhile, both zinc deficiency and copper deficiency induced by zinc overload decrease Zn/Cu‐SOD activity.^[^ ^92^, ^93^ ^]^ Therefore, both zinc depletion and rapid zinc overload damage mitochondria, promote ROS production, and trigger apoptosis induced by oxidative stress stimulators in leukemia.^[^ ^9^, ^94^ ^]^

#### 4.2.4 Modulation of Leukemia Cell Differentiation by Zinc

Zinc deficiency decreases the production of red blood cells and lymphocytes while increasing the production of granulocytes and monocytes.^[^ ^95^ ^]^ The role of zinc signaling in leukemogenesis is similar to its role in physiological hematopoiesis (Figure 5).

Zinc inhibits cAMP‐mediated myelomonocytic leukemia differentiation by inhibiting adenylate cyclase activity.^[^ ^96^, ^97^, ^98^ ^]^ Although there is no definitive evidence for the role of zinc in lymphoid leukemia or lymphoma differentiation, it has been suggested that zinc reduces IL‐4‐mediated expression of CD5 in B‐lymphoid leukemia cells, thereby preventing leukemia cells from becoming apoptosis tolerant.^[^ ^99^ ^]^

Zinc regulates the Myc network to decide the differentiation direction for myeloid leukemia by reducing c‐Myc and promoting Mad1 in leukemia. While the member of the Myc network, Mxi and Mad1, turns the differentiation to erythroid direction, c‐Myc turns the direction to myelomonocytic differentiation.^[^ ^100^, ^101^ ^]^ Zinc downregulates c‐Myc but upregulates Mad1, suggesting its importance in promoting erythroid differentiation, which benefits the therapy of erythroleukemia rather than myelomonocytic leukemia.^[^ ^102^ ^]^ Overall, zinc appears to promote erythroid and lymphoid differentiation, but suppresses myelomonocytic differentiation, thus increasing the risk of myeloid leukemia.

Over hundreds of zinc finger proteins have been found to be associated with hematopoiesis, contributing to the development of both myeloid and lymphoid lineages.^[^ ^103^ ^]^ Most zinc finger proteins act as the transcription factors leading to the expression of downstream genes. Some zinc finger proteins, such as ZFP36L1/ZFP36L2, lead to the degradation of target mRNA, thereby maintaining physiological differentiation and proliferation. Aberrant expression of zinc finger proteins is common in leukemia, including rearrangements, overexpression, abnormal epigenetic modification and mutations, which are also associated with leukemogenesis (**Table** **2**). Zinc finger domains in these proteins are critical for DNA binding and partner protein recruitment, and most are essential for leukemia development. How to treat leukemia by targeting zinc homeostasis and thus modulating ZFPs deserves further in‐depth studies in the future.

**Table 2:** Zinc Finger Proteins Associated with Leukemia.

Zinc finger protein	References	Leukemia‐related aberrant expression	Role of zinc finger domains in leukemia
ZBTB46	[104, 105]	ZBTB46 is highly expressed in AML and is associated with a poor prognosis.	There is no clear evidence linking the zinc finger domain of ZBTB46 to leukaemia.
GLIS2	[106, 107, 108]	The translocation (inv(16)(p13.3q24.3)) involving CBFA2T3 and GLIS2 occurs in 20–30% of non‐DS‐AMKL and is associated with a poor prognosis.	The DNA binding domain (DBD) of GLIS2 contains five zinc finger motifs and is essential for megakaryocyte differentiation.
EHZF	[109, 110, 111]	Some AML and CML patients express high levels of EHZF and are thought to be strongly associated with MLL rearrangement.	Carboxyl‐terminal zinc fingers of EHZF mediate the interaction of EBF1 to induce B‐lymphoid differentiation.
MOZ	[112]	The translocation t(8;16)(p11;p13) resulting in the MOZ‐CBP fusion gene, t(8;22)(p11;q13) resulting in the MOZ‐p300 fusion gene, inv(8)(p11;q13) resulting in the MOZ‐TIF2 fusion gene is present in AMLs and leads to aberrant acetylation for transcriptional regulation and leukemogenesis.	Both the C2HC zinc finger motif and the Myst sequence in the HAT domain are required for AML initiation. Zinc finger in plant homeobox‐like domain also contributes to AML development.
IKAROS	[113, 114]	The disrupted mutation of IKAROS is present in ALL, CML and lymphomas and has been identified as a leukemia predisposition gene.	Kruppel‐type zinc finger domains in both the C and N termini are required for IKAROS function and prevention of T‐cell leukemia and lymphoma.
EVI‐1	[115]	The t(3;21)(q26;q22) translocation resulting in AML1‐EVI1 occurs in AML and MDS and leads to leukemogenesis. High EVI‐1 expression was an independent negative prognostic indicator for survival in AML patients.	Both the N‐terminal region (7 zinc finger domains) and the C‐terminal region (3 zinc finger domains) contribute to leukemogenesis.
GATA2	[116, 117, 118]	Overexpression of GATA‐2 is also implicated in leukemic disease and is an indicator of poor prognosis AML. In addition, the N‐terminal frameshift mutations and mutations in the first and second zinc fingers of GATA2 are presented in AML and MDS.	The zinc fingers of GATA2 are critical for both DNA binding and interactions with partner proteins for its normal physiological function, which leads to AML and MDS upon zinc finger loss or mutation.
GATA1	[116]	GATA1 mutations are normal in transient myeloproliferative disorder and acute megakaryoblastic leukemia in children with Down syndrome (DS), leading to leukemogenesis.	The N‐terminal zinc fingers of GATA1 are essential for DNA binding and FOG‐1 recruitment, which are required for normal hematopoiesis. Mutation of the N‐terminal zinc fingers of GATA1 results in myeloid malignancies in children with DS.
GATA3	[119, 120]	GATA3 variants are associated with ALL susceptibility and risk of relapse, possibly via CRLF2 modulation.	No clear evidence exists linking the zinc finger domain to leukemogenesis.
PLZF	[121, 122]	The t(11;17) (q23;21) chromosomal translocation occurs in about 2% of all APL, in which PLZF is fused to the RARα gene on chromosome 17, forming a fusion protein.	Zinc is required for the stability of PLZF. The POZ (Pox virus and zinc finger) domain at the N‐terminal of PLZF is essential for transcriptional repression, while nine C2H2‐type zinc fingers at the C‐terminal of PLZF play an important role in the nuclear‐cytoplasmic reaction. The onco‐fusion protein PLZF‐RARα or RARα‐PLZF retains only POZ‐BTB and 2 C2H2 zinc fingers or 7 zinc fingers, resulting in disruption of PLZF function in hematopoiesis.
ETO	[123, 124, 125, 126]	The t(8;21)(q22;q22) translocation mediates the expression of the fusion protein AML1‐ETO, which typically occurs in the M2 subtype of AML. Patients with t(8;21) AML have a relatively favorable prognosis, but are prone to relapse.	ETO is involved in transcriptional repression through the recruitment of histone deacetylase complexes. Among these, the NHR4 domain containing the MYND class of zinc fingers is proposed to interact with SON and N‐CoR to trigger signals that inhibit leukemogenesis.
ZNF384	[127, 128]	ZNF384 rearrangement is common in ALL. High expression of ZNF384 is associated with poor survival in MLL and B‐ALL. Several ZNF384 fusions have been identified, including EP300‐ZNF384, CREBBP‐ZNF384, TCF3‐ZNF384, TCF4‐ZNF384, TAF15‐ZNF384, and EWSR1‐ZNF384.	The C2H2 type zinc finger in ZNF384 plays a role in integrin binding and p130Cas binding. However, there is no clear evidence of a relationship between the zinc finger domain and leukemogenesis.
ZEB1	[129, 130]	ZEB1 is frequently disrupted in adult T‐cell lymphoma/leukemia cells, which is associated with leukemogenesis.	Loss of function of the C‐terminal zinc finger cluster leads to CD4^+^ T‐cell lymphoma.
ZEB2	[129, 131]	The t(2;14)(q22; q32) translocation involving the ZEB2 and BCL11B loci has been identified in early T‐cell precursor ALL. Overexpression of ZEB2 is associated with spontaneously developing immature T‐ALL.	No clear evidence exists linking the zinc finger domain of ZEB2 to leukemogenesis.
ZFP36L1/ZFP36L2	[132, 133, 134]	Depletion of ZFP36L1 and ZFP36L2 leads to T‐ALL after impaired thymic development.	The ZFP36 family of proteins contain two tandemly repeated CCCH‐type zinc finger motifs, bind to adenine‐uridine‐rich elements in the 3′ UTR of specific mRNAs, and cause target mRNA degradation to maintain the marginal zone B‐cell compartment or monocyte/macrophage differentiation of hematopoietic stem/progenitor cells.
ZNF300	[135]	ZNF300 methylation increases in MDS and AML.	No clear evidence exists linking the zinc finger domain of ZNF300 to leukemogenesis.
ZNF224	[136, 137, 138]	ZNF224 expression increases during CLL progression and predicts a poor prognosis.	ZNF224 acts as a transcriptional repressor to inhibit oncogene such as c‐Myc and AXL to promote apoptosis. However, no clear evidence shows the relationship between its zinc finger domain and leukemogenesis.

### 4.3 Crosstalk between Zinc and Other Metal Elements

A recent study based on metallomics has investigated the systematic disturbance of serum metal ions in leukemia development.^[^ ^139^ ^]^ Zinc supplementation was used to alleviate copper overload‐mediated hypercupremia, suggesting the efficacy of zinc in leukemia treatment by affecting the homeostasis of other metal and semimetal ions.^[^ ^140^ ^]^

Zinc overload is predicted to prevent the uptake of most divalent cations due to competition for divalent cation channels. Compelling evidence shows the antagonistic relationship between zinc and arsenic, copper, cadmium, selenium, and iron.^[^ ^141^, ^142^, ^143^, ^144^ ^]^ Among these metals and semimetals, arsenic is widely used in APL, CML, and adult T‐cell leukemia, but has been found to compete with zinc for both uptake and protein binding, suggesting the potential risk of zinc supplementation in arsenic‐involved therapy.^[^ ^145^, ^146^ ^]^ The zinc homeostasis‐related proteins ZnT1 and metal regulatory transcription factor 1 (MTF1) also increased the resistance of CML to arsenic trioxide.^[^ ^147^ ^]^ On the other hand, zinc increased the sensitivity of cis‐dichlorodiammine‐platinum‐resistant lymphoid leukemia to cadmium, which may be synergistically applied in platinum‐resistant lymphoid leukemia.^[^ ^148^ ^]^ We also noted that divalent cations such as iron and copper competed with zinc and were associated with more aggressive forms of leukemia, whereas elevated levels of selenium and magnesium were associated with improved response to leukemia therapy.^[^ ^139^, ^149^, ^150^ ^]^

## 5 Zinc‐Based Therapeutic Strategies for Leukemia

**Figure 6:**
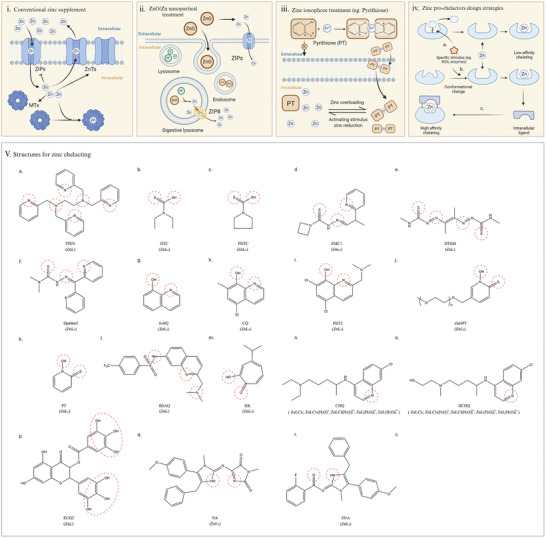
Potential Strategies for Zinc Regulation in Leukemia Therapy. Zinc supplementation requires channel‐mediated transport to ensure controlled zinc influx and feedback regulation of homeostatic proteins to prevent toxic accumulation in leukemia cells (i). Toxic zinc levels can occur through endocytosis‐mediated uptake of Zn nanoparticles and upregulation of ZIP8 in lysosomes, leading to rapid influx and oxidative stress (ii). Ionophores can facilitate zinc transport across membranes and release zinc ions in response to deficiency or specific stimuli (iii). Pro‐chelators promote zinc depletion by various mechanisms, including blocker removal (iv, a), molecular switches (iv, b), and affinity enhancement (iv, c). The structures of eighteen zinc‐binding molecules are shown (v, a‐r), with key binding groups highlighted in red circles. Typically, zinc forms a tetrahedral structure with ligands in a 1:2 ratio, while TPEN, DTSM, BSAQ, and ECGC bind zinc in a 1:1 ratio. CHQ and HCHQ chelate zinc with H2O or Cl‐ to form five‐coordinate complexes.

Systemic zinc depletion can lead to adverse side effects and a poor prognosis. Conversely, traditional zinc supplementation has been moderate, potentially protecting leukemia cells from apoptosis and thus reducing therapeutic efficacy. Rapid elevation of intracellular zinc levels or precise zinc depletion may prove beneficial in the context of limiting leukemia progression. Potential zinc modulation strategies for leukemia therapy are highlighted in **Figure** **6**i‐iv.

### 5.1 Zinc‐Binding Molecules for Intracellular Zinc Modulation

The hydrophilic nature of zinc ions typically dictates their transport through channel proteins, where hydrophobic molecules facilitate the zinc transmembrane process and achieve rapid modulation of intracellular zinc. The use of chelators and ionophores (Figure 5v) to affect metal homeostasis is considered a promising strategy.

Zinc is known to prefer ligands such as thiones, sp2 hybridized nitrogens, and phenoxides, which serve as moderate donors.^[^ ^151^ ^]^ Less specific ligands include sp3 hybridized nitrogens and polyphenol structures, with water molecules also contributing to zinc chelation, although to a lesser extent.^[^ ^152^ ^]^ It is important to note that the zinc‐binding structures mentioned above have a higher affinity for copper than for zinc, suggesting that the presence of copper may influence the efficiency of zinc ionophores or chelators.^[^ ^151^ ^]^

#### 5.1.1 Zinc Ionophores for Zinc Supplementation

Zinc ionophores are lipophilic ligands that reversibly bind to metal cations and release the metal ions in response to low intracellular metal concentrations or specific conditions.^[^ ^151^, ^153^ ^]^ Therefore, the efficacy of a zinc ionophore is determined by its binding affinity and the distribution of zinc within leukemia cells.^[^ ^151^ ^]^ Typically, non‐specific metal binding sites on proteins have a dissociation constant (Kd) greater than 10^−6^ m, whereas metalloproteins have Kd values below 10^−7^ m, suggesting that ionophores should have Kd values below 10^−6^ m but higher than those of potential zinc acceptor proteins.^[^ ^154^ ^]^ Ionophores such as Dp44mT, PBT2, and epigallocatechin gallate (ECGC) have lower affinities for zinc than serum albumin, suggesting that they may release zinc ions systemically rather than specifically in leukemia cells.^[^ ^155^, ^156^, ^157^ ^]^ In contrast, ligands such as 8‐hydroxyquinoline (8‐HQ), chloroquine (CQ), pyrrolidine dithiocarbamate (PDTC), and pyrithione have the appropriate affinity for zinc to transport into the cytoplasm.^[^ ^153^ ^]^

Rapid zinc accumulation, synergistic with various ligands, exerts anti‐leukemic activity through multiple mechanisms. Pyrithione, an FDA‐approved antifungal drug, has been shown to induce apoptosis and inhibit proteasome‐associated deubiquitinating enzymes in leukemia, potentially providing superior therapeutic effects to zinc alone.^[^ ^158^, ^159^ ^]^ To address the poor solubility of pyrithione zinc, Sessler et al. developed a water‐soluble form with oligoethylene glycol chains attached to the pyrithione rings.^[^ ^160^ ^]^ CQ has also been shown to increase intracellular zinc levels and inhibit tumor growth, possibly through zinc‐mediated binding to histone deacetylases.^[^ ^161^ ^]^ Other classical zinc ionophores such as diethyldithiocarbamate (DTC), PDTC, ZNA, ZMC1, and 8‐HQ have been investigated for anti‐tumor activity, although their specific efficacy in leukemia remains to be established.^[^ ^151^, ^162^ ^]^

#### 5.1.2 Chelator and Pro‐Chelator for Zinc Depletion

The therapeutic potential of zinc depletion in leukemia has mainly led to the exploration of chelation strategies. The zinc chelator TPEN is widely used to create zinc‐deficient conditions for preclinical research, while clinical evidence of its pharmacokinetic properties is lacking. Notably, TPEN is also non‐specifically affinite to copper, calcium and magnesium, suggesting that metal rebound experiments are required for the TPEN‐mediated zinc deficiency model.

Systemic zinc deficiency induced by a low‐zinc diet can impair immune function in leukemia patients, increasing several health risks. Given the potential for increased side effects, it is critical to develop novel zinc depletion strategies that specifically target leukemia cells without causing systemic deficiency.

To minimize off‐target toxicity, reversible chelators have been modified to reduce their binding activity in response to specific pathological stimuli, known as pro‐chelators. Three main strategies have been used in the design of pro‐chelators: removal, switching and addition (Figure 6‐iv.).^[^ ^163^ ^]^ The removal strategy involves a removable group that blocks the zinc binding site and can be released under conditions of oxidative stress, such as the boronic acid pinacol ester group.^[^ ^164^ ^]^ The switching strategy alters the molecular configuration to achieve the optimal position for metal binding under specific stimuli, using UV radiation to induce changes in molecular structure.^[^ ^165^ ^]^ Structures such as azobenzene, which are sensitive to photochromic stimulation, are also suitable for pro‐chelator design using a switching strategy.^[^ ^166^ ^]^ The addition strategy incorporates a specific element in the pathological state that increases the thermodynamic stability of the metal‐ligand complex, which may be critical for the design of pro‐chelators in the context of leukemia.^[^ ^163^ ^]^

### 5.2 Zinc Nanoparticles in Leukemia Therapy

While zinc nanoparticles are partially solubilized extracellularly and enter with a traditional transporter‐mediated mode, most nanoparticles are completely internalized and release zinc ions under acidic conditions in the lysosome.^[^ ^167^ ^]^ ZIP8, the zinc channel loading lysosomal zinc transmembrane transport, is highly expressed in AML cells and allows a rapid increase of free zinc in the cytoplasm during zinc nanoparticle treatment.^[^ ^56^, ^168^ ^]^ Both zinc oxide and zinc sulfide are common forms of solid zinc used for nanoparticle preparation and have been shown to increase ROS production in leukemia cells.^[^ ^169^, ^170^ ^]^ Synthesized by various methods, zinc nanoparticles can assume multiple morphologies, including quantum dots, spheres, rods, and tetrapods, each with different pharmacokinetic properties and targeting capabilities, allowing for a wide range of therapeutic strategies.^[^ ^171^ ^]^

### 5.3 Modulation of Zinc Transportation for Leukemia Therapy

While the channel‐based model is a majority in zinc trans‐membrane transport, the modulation of zinc transporters is proposed for the species regulation of subcellular zinc distribution. We highlight the highly expressed ZIP11 in AML, which is located in the nucleus for nuclear zinc release, leads to the low nuclear zinc level, and should be inhibited for leukemia therapy.^[^ ^56^ ^]^ ZIP8, which is expressed on lysosomes, plays a role in promoting zinc release, potentially benefiting the zinc nanoparticle‐involved strategy.

Given the role of zinc signaling in leukemia cell differentiation and survival, targeting the ER‐localized zinc transporters ZIP7 and ZnT7 may be a potential strategy for FZS and ER stress modulation.^[^ ^172^ ^]^ Fryer et al. identified NVS‐ZP7‐4 as a potent ZIP7 inhibitor by phenotypic screening. NVS‐ZP7‐4 induces zinc accumulation and ER stress and induces apoptosis in T‐ALL cells.^[^ ^173^ ^]^

We also highlight that homologous peptides such as phytohemagglutinin and poly‐L‐ornithine have been found to inhibit transferrin‐bound zinc uptake in CML with higher efficiency than in normal lymphocytes and further influence the subcellular distribution of zinc.^[^ ^174^, ^175^ ^]^

Generally, a deeper understanding of zinc transporters and the subsequent development of various zinc transporter agonists and antagonists will help to realize the development of novel leukemia therapeutic strategies by precisely regulating zinc homeostasis at the subcellular level.

### 5.4 Potential Role of Zinc in Immunotherapy for Leukemia

**Figure 7:**
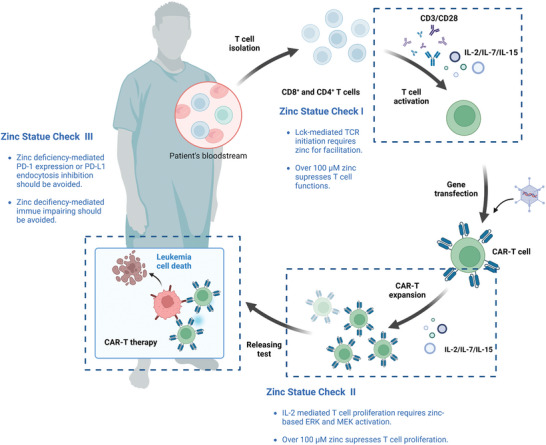
The Role of Zinc in CAR‐T Therapy. We propose three key checkpoints where zinc status affects CAR‐T cell therapy, aiming to optimize cell‐based immunotherapy. T‐cell proliferation and activation depend on adequate zinc levels; however, excess zinc can impair T‐cell function. Thus, precise zinc regulation is crucial during the ex vivo activation and expansion of T cells (Zinc Checkpoint I and II). Zinc dysregulation is common in several leukemias, often resulting in reduced zinc levels following chemotherapy. We note that zinc deficiency can impair hematopoietic organs such as the thymus and promote immune evasion mechanisms such as PD‐L1 upregulation, potentially leading to resistance to immunotherapies (Zinc Checkpoint III).

The absence of physical tumor barriers has made cell‐based immunotherapies remarkably effective in the treatment of hematologic malignancies.^[^ ^176^ ^]^ Allogeneic hematopoietic stem cell transplantation (HSCT), the earliest form of immunotherapy used in AML, confers a superior likelihood of achieving long‐term, durable remission compared to conventional chemotherapy.^[^ ^177^ ^]^ A phase II clinical trial suggested that high‐dose oral zinc supplementation enhances immune reconstruction, particularly for thymic reconstitution after HSCT, improves thymic performance, and reduces the risk of infection in elderly leukemia patients.^[^ ^178^ ^]^ Recently, antibody‐based modalities, including chimeric antigen receptor (CAR) T cells and antibody‐drug conjugates, have gained widespread recognition in the treatment of ALL and AML, particularly in B‐cell leukemia, due to the limited expression of CD19 or CD20 antigens on leukemic blasts.^[^ ^179^ ^]^ We propose a hypothesis of three zinc checkpoints in CAR‐T therapy, where zinc exerts key efficacy for T‐cell production and prevention of immune evasion, potentially benefiting CAR‐T treatment overcome T‐cell production and immune evasion challenge, promoting further impede clinical translation (**Figure** **7**).

#### 5.4.1 Zinc in CAR‐T Cell Production

The optimization of ex vivo stimulation and expansion conditions for isolated T cells is essential for the production of CAR‐T cells.^[^ ^180^ ^]^ We have proposed ex vivo stimulation and expansion as zinc checkpoints I and II for the zinc dependence of T cell activation and proliferation. T cell activation requires the T cell receptor (TCR) to recognize properly presented antigenic peptides and to transduce the signal intracellularly via CD3. Zinc is present in the C‐terminal region of CD4/CD8α and the N‐terminal of lymphocyte‐specific protein tyrosine kinase (Lck), forming a “zinc clasp” structure that facilitates the interaction between Lck and CD4/CD8. This interaction brings lymphocyte‐specific Lck into proximity with the TCR complex, initiating a cascade of events including phosphorylation of immunoreceptor tyrosine‐based activation motifs and zeta‐chain‐associated protein kinase 70, thereby activating the TCR signaling pathway.^[^ ^181^, ^182^ ^]^ Since the TCR complex lacks intrinsic kinase activity and requires Src family tyrosine kinases such as Lck for signaling, the concentration of zinc in the culture milieu is critical for CD3/CD28‐mediated T cell activation.^[^ ^63^ ^]^ Adequate intracellular zinc is also required for IL‐2‐mediated phosphorylation of MEK and ERK1/2, which are essential for T cell proliferation.^[^ ^183^ ^]^ While IL‐2, IL‐7, or IL‐15 are commonly used to optimize expansion conditions, adequate zinc supplementation is essential to maintain the proliferative potential of T cells. However, excessive zinc (above 100 µm) in culture can inhibit T cell activation and proliferation via IL‐1 by blocking the activity of IL‐1 receptor‐associated kinase (IRAK).^[^ ^184^ ^]^ Zinc concentrations can be tightly controlled in vitro, so an appropriate low‐concentration zinc supplement should be considered to optimize activation or expansion conditions.

#### 5.4.2 Zinc in Immune Checkpoint Blockade

Upregulation of immune checkpoint ligands is a key strategy used by leukemic blasts to evade detection by T cells and NK cells. In particular, increased expression of PD‐1 has been observed on CD8^+^ T cells and regulatory B cells in the bone marrow of AML patients, which is strongly correlated with poor prognosis.^[^ ^185^, ^186^ ^]^ Zinc supplementation decreases PD‐L1 expression in NSCLC, possibly through endocytosis of surface EGFR.^[^ ^187^ ^]^ A similar endocytic mechanism has been observed in hepatocellular carcinoma‐associated macrophages, where zinc also decreased surface PD‐L1 expression.^[^ ^188^ ^]^ The decreased expression of PD‐L1 in response to zinc supplementation alleviates the depletion of PD‐1‐expressing lymphocytes derived from both tumor and innate immune compartments.

Killer cell inhibitory receptors (KIRs), which contain a zinc‐binding motif for HLA recognition, are zinc‐dependent for the inhibition of NK cytotoxicity.^[^ ^189^ ^]^ In the context of leukemia, where loss or reduction of HLA heterozygosity or complete loss of an HLA haplotype may occur, the cytotoxic potential of NK cells may not be significantly inhibited by zinc supplementation.^[^ ^190^ ^]^ The use of zinc intervention in NK‐mediated immunotherapy warrants careful consideration.

#### 5.4.3 Zinc and the Tumor Immune Microenvironment (TME) in Leukemia

The strategy of targeting TME is considered one of the most important directions to overcome the refractory or intolerant problem in conventional treatment of leukemia and lymphoma.^[^ ^191^, ^192^, ^193^ ^]^ Stromal cells and various immune cells, including tumor‐associated macrophages (TAMs), tumor‐associated neutrophils (TANs), myeloid‐derived suppressor cells (MDSCs), and tumor‐associated dendritic cells (TADCs), are crucial in suppressing antitumor immunity in leukemia. Collectively, these cell types are emerging as promising targets for therapeutic intervention.

While zinc is suggested to assist the maturation and activation of both innate and adaptive immunity, zinc supplementation might be essential for promoting the TME in leukemia therapy.^[^ ^194^, ^195^ ^]^ Studies have indicated that zinc supplementation via Zinc‐L‐carnosine can reduce the levels of immunosuppressive cells such as Treg, MDSC, and M2 macrophages, and enhance CD8^+^ T cells in colorectal cancer, suggesting a positive effect of zinc in solid tumors.^[^ ^196^ ^]^ Conversely, matrix metalloproteinases (MMPs), which are zinc‐dependent enzymes, have been found to induce leukemic cell growth, migration, invasiveness, and angiogenesis, and are associated with the progression of malignancies through their degradative activities.^[^ ^1^, ^197^ ^]^ Zinc chelation may inhibit the invasion of leukemia cells via inhibiting MMP activity.^[^ ^198^ ^]^ T zinc finger protein A20, a positive regulator for MDSC survival, can be upregulated by zinc supplements, which implies that zinc deprivation might benefit the repression of MDSCs.^[^ ^199^, ^200^ ^]^ Therefore, zinc homeostasis may play a “double‐edged sword” role in the immune microenvironment of leukemia, and how to remodel the anticancer immune microenvironment by influencing zinc homeostasis under different conditions deserves further investigation.

## 6 Conclusion and Future Prospects

**Figure 8:**
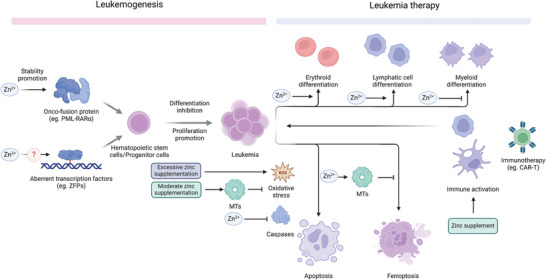
Implication of Zinc Ions in Leukemia. The effect of zinc in onco‐fusion proteins and transcription factors is associated with leukemia progression. The effect of zinc on immune development/activation and PCD regulation implies its potential therapeutic application.

Disruption of systemic zinc distribution has been observed in patients with leukemia, highlighting the clinical potential of zinc in the diagnosis, treatment, and prognosis of this disease. Further investigation of specific intracellular zinc distribution is warranted, as zinc signaling may play a critical role in leukemia progression by influencing the accumulation of PML‐RARα and BCR‐ABL, as well as modulating processes such as differentiation, apoptosis, and ferroptosis (**Figure** **8**).

Despite the complex role of zinc in health maintenance and leukemia development, zinc supplementation has been shown to improve patient quality of life, but does not appear to improve leukemia‐related biomarkers. Conversely, zinc depletion can induce apoptosis in leukemia cells, but this can also adversely affect normal tissues, resulting in a higher mortality rate. Managing zinc levels is critical in the context of cell‐based immunotherapy for leukemia.

To advance leukemia therapy, there is an urgent need for novel strategies targeting zinc transport and precise regulation of zinc signaling. The various zinc transporters could serve as potential targets for targeted modulation of zinc signaling at the subcellular level. In addition, the development of safer and more effective zinc carriers, chelators, and zinc‐related nanomaterials holds great promise for future clinical applications in the treatment of leukemia.

## Conflict of Interest

The authors declare no conflict of interest.

## Author Contributions

B.Z., C.Y., and S.H. contributed equally to this work. B.Z., Q.Z., and Z.H. conceptualized this study. Z.H. funded the study. B.Z., C.Y., and S.H. wrote the main manuscript, and B.Z., Q.Z., S.H., and Z.H. reviewed and edited the text. B.Z. and C.Y. designed and generated all the pictures. P.S., K.L., W.Q., S.X., and Z.L. helped sort out the literature. All authors read and approved the final manuscript.
